# Vaginal dose of radical radiotherapy for cervical cancer in China: a multicenter study

**DOI:** 10.1186/s12885-019-6423-5

**Published:** 2019-12-16

**Authors:** Juan Wang, Kai-shuo Zhang, Tao Wang, Zi Liu, Rui-hua Wang, Fu-quan Zhang, Lang Yu, Li Ran, Jian-li He, Ya-li Wang, Li-chun Wei, Mei Shi, Guo-qing Wang, Chao-qun Wu, Qi-jun Kang, Jie Yang, Sha Li, Fei-yue Yang, Bao-gang Liu, Juan-yue Liu, Fan Shi, Jin Su, Wei Yuan, Emmanuel Kwateng Drokow

**Affiliations:** 1 Department of Radiation Oncology, The First Affiliated Hospital of Xi’an Jiao Tong University, 277# West Yanta Road, Xi’an, Shaanxi China; 2 Department of Radiation Oncology, Peking Union Medical College Hospital, Chinese Academy of Medical Sciences and Peking Union Medical College, Beijing, 100032 China; 3 Department of Oncology, The Affiliated Hospital of Guizhou Medical Universty/Guizhou Cancer Hospital, Gui Yang, 550000 China; 4 Department of Radiation Oncology, General Hospital of Ningxia Medical University, Yin chuan, 750004 China; 5 Department of Radiation Oncology, The Second Affiliated hospital of Xi’an Jiao Tong University, Xi’an, 710004 China; 6 Department of Radiation Oncology, First Affiliated Hospital of Air Force Medical University, Xi’an, 710032 China; 7 Department of Gynecology Oncology, Shaanxi Provincial Tumor Hospital, Xi’an, 710061 China; 8 Department of Radiation Oncology, The first people’s Hospital of Kashi District, Xinjiang, 844000 China; 9 Department of Radiation Oncology, People’s Hospital of Xinjiang Uygur Autonomous Region, Urumchi, 830002 China; 10 Department of Radiation Oncology, The 940th Hospital of Joint Logistics Support force of Chinese People’s Liberation Army, Lan Zhou, 730050 China; 11 Department of Oncology, Guizhou Provincial People’s Hospital, Guiyang, 550002 China; 12 Department of Radiation Oncology, The Second Affiliated Hospital of Shaanxi University of Chinese Medicine, Xian yang, 712046 China; 13 Department of Radiation Oncology, Xi’an Gao Xin Hospital, Xi’an, 710000 China

**Keywords:** Cervical cancer, Radiotherapy, Vaginal dose, PIBS

## Abstract

**Background:**

The posterior-inferior border of symphysis (PIBS) point system is a novel vaginal dose-reporting method and is a simple and reliable method proposed by the Medical University of Vienna proposed for both external-beam radiotherapy (EBRT) and brachytherapy (BT). In this multicenter study, we sought to first evaluate the vaginal radiation dose in Chinese cervical cancer patients according to the PIBS point system and then to analyze the factors influencing the dose distribution.

**Methods:**

We collected data from the medical records of 936 cervical cancer patients who underwent concurrent radiochemotherapy at 13 different institutions in China. Radiation doses at points A, PIBS+ 2 cm, PIBS and PIBS-2 cm, International Commission on Radiation Units (ICRU)-R and ICRU-B were measured.

**Results:**

The median total doses in EQD2_α/β = 3_ at points PIBS+ 2 cm, PIBS and PIBS-2 cm were 82.5 (52.7–392.1) Gy, 56.2 (51.4–82.1) Gy and 2.6 (0.9–7.4) Gy, respectively. The median total doses in EQD2_α/β = 3_ at ICRU-R and ICRU-B were 77.5 (54.8–132.4) Gy and 79.9 (60.7–133.7) Gy, respectively. The mean vaginal reference length (VRL) was 4.6 ± 1.0 cm (median, 4.5 cm). In patients with VRL ≤4.5 cm, the mean total doses in EQD2_α/β = 3_ at points PIBS+ 2 cm, PIBS and PIBS-2 cm were 128.5, 60.7 and 0.8 Gy, respectively. In patients with VRL > 4.5 cm, the mean total doses at these three points were 68.9, 0.5 and 54.5 Gy, respectively. Classification of patients revealed significant differences (*P* < 0.05) between these two groups.

**Conclusions:**

With the PIBS point system, Chinese patients with a shorter VRL of < 4.5 cm received higher radiation doses at the PIBS+ 2 cm, PIBS and PIBS-2 cm points than European and American patients. Further studies are required to establish the dose–effect relationships with these points as references.

The study was registered as a clinical trial (NCT03257475) on August 22, 2017.

## Background

Locally advanced cervical cancer, which is classified as stage ≥IB2 as per the International Federation of Gynecology and Obstetrics (FIGO) criteria, is treated with a concurrent radiochemotherapy. Radiotherapy involves the administration of pelvic external-beam radiotherapy (EBRT) as well as brachytherapy (BT). However, in cases of locally advanced cervical cancer, the upper part of the vagina is considered a target organ for radiotherapy, which puts the higher part of the vagina at risk of radiotherapy-induced injury. Despite this, investigations on vaginal morbidity after radiotherapy have been scarce.

Our previous analysis on vaginal morbidity in cervical cancer patients treated with the current treatment strategies revealed that the incidence of vaginal radiation injury was 84.4% (238/282), and the incidence rates of radiation injury of grades II, and III were 29.8% (84/282) and 3.9% (11/282, *P* < 0.05), respectively [1]. Kirchheiner et al. found that vaginal morbidity occurred in 255 of 630 (40.5%) patients, with morbidity rates for grades II and III being 17, and 1%, respectively [2]. Similarly, Susko et al. found that of the morbidity rates for grades II and III or higher vaginal toxicity were 41.7 and 17.6%, respectively, in 36 of 62 cervical cancer or uterine cancer patients [3]. Multi-factor analysis indicated that the dose of BT was an independent factor affecting the occurrence of vaginal radiation-induced injury (*P* = 0.043) [1].

To identify a simple and reliable representation of the radiation exposure in the vagina and establish a method suitable for both EBRT and BT, the Medical University of Vienna proposed a novel vaginal dose-reporting method [4]. This method allows for the determination of the total (EBRT+BT) dose (EQD2) administered to the upper, mid, and lower parts of the vagina. Further, this evaluation system can be used for 2D and 3D BT. In this study, we sought to test the applicability of this method in Chinese patients and determine the vaginal dose administered in patients, with a view to identifying the influencing factors for vaginal radiation injury in the future.

## Methods

This investigation was designed as an observational study of patients who underwent concurrent radiochemotherapy at any of the 13 participating hospitals between December 2016 and June 2018. The study was registered as a clinical trial (NCT03257475) and approved by the ethics committee of the First Affiliated Hospital of Xi’an Jiaotong University (No. XJTU1AF2017LSK-11). Informed consent was obtained from all patients who participated in this study.

In all, we selected 936 of the 1194 cases, for which complete data were available, excluded 30 patients for lack of complete clinical information and exclued 228 patients aged > 60 years, since elasticity and length of the vagina are affected by estrogen level. All patients were diagnosed with cervical carcinoma of stages IIA1 to IVB (except for IIIA), classified as per the 2009 FIGO criteria [5]. Twelve patients who had enlargement of para-aortic lymph nodes received extended field radiation. EBRT was delivered to the entire pelvis at 1.8–2.0 Gy per fraction, for a total of 25 fractions and 45–50 Gy (3D-conformal radiotherapy [CRT] or intensity-modulated radiotherapy [IMRT]), with concurrent weekly administration of cisplatin (25 mg/m^2^, [6] or cisplatin (25 mg/m^2^, day 1–day 3) combined with liposome paclitaxel (135 mg/m^2^, day 1). The target was contoured according to recommendations of the Radiation Therapy Oncology Group (RTOG) with three-dimensional (3D) computed tomography (CT)-assisted treatment planning [7].

High-dose rate (HDR) BT was administered at doses of 24 or 30 Gy in 4 to 5 fractions after EBRT using the Fletcher-Suit-Delclos set with a microSelectron HDR (Elektra Brachy, Sweden).The planning aim at point A was 82–90 Gy in EQD2. BT was administered via the CT/simulation (CT/SIM)-guided technique with a tandem-ovoid applicator. A CT/SIM scan of the pelvis was performed in the first and third BT fractions. The obtained images were transferred onto Oncentra TPS. For treatment planning, the point A dose-evaluation method was used, with the loading dose based on the Manchester system of BT, with the objective of delivering 6 Gy, which at EQD2 was 8 Gy to point A. A sum of the BT and EBRT doses was determined applying the linear-quadratic model, with an α/β ratio of 10 Gy.

### Data collection and definitions

The PIBS point proposed by Westerveld et al. [4], was defined in the sagittal images. A virtual anteroposterior line was drawn at the level of the inferior border of the symphysis to cross the vertical axis of the uterine tandem, and the following points were marked: PIBS+ 2 cm and PIBS-2 cm were defined 2 cm cranial and caudal to the PIBS, respectively. The point PIBS+ 2 and PIBS-2 were considered to represent the anatomical middle and introitus part of the vagina [4]. The vaginal reference length (VRL) was defined as the distance from the center of the vaginal sources to the PIBS point.

The rectal (rectovaginal) point and bladder point were also plotted as per the guidelines of International Commission on Radiation Units (ICRU) Report 89 and ICRU Report 38 [8, 9].

Patients underwent a CT scan at the time of the first (BT1) and third (BT3) fractions of BT. BT2, 4 and 5’s parameters recorded by taking the dose of average values of BT1 and BT3.

### Statistical analysis

The doses were represented in terms of EQD2, as described above. The SPSS statistical software system (version 18, SPSS, Inc., USA) was used for all statistical analyses. Descriptive statistics were used to indicate mean, median, standard deviation, and range.

## Results

### Demographic and clinical characteristics of the study population

The demographic and clinical features of the 936 patients are listed in Table 1. Most patients had lesions of stages IIB (455/936, 48.6%) or IIIB (243/936, 26.0%). Almost all patients (929, 99.2%) had squamous cell carcinoma; the remaining seven had adenocarcinoma (0.8%). Of the 936 patients, 83 (8.8%) had vaginal involvement (residual disease) at the time of BT, as determined by clinical examination and imaging studies. Most of the residual lesions were localized to the vaginal fornix, or some patients with bulky disease still had residual disease in the vagina after EBRT. The mean VRL was 4.6 ± 1.0 cm (median, 4.5 cm; range, 1.3–8.4 cm). The distance between two vaginal applicators was 2.8 ± 0.4 cm (range, 1.8–4.1 cm). The length of the intrauterine catheters was 6.0 ± 0.6 cm (range, 3.7–8.5 cm).

**Table 1:** Patient characteristics (*N* = 936)

Characteristics	Value
Mean age (range)	50 (25–60) y
FIGO stage (n, %)	
IB1	6 (0.6)
IB2	66 (7.1)
IIA1	33 (3.5)
IIA2	94 (10.0)
IIB	455 (48.6)
IIIB	243 (26.0)
IVA	8 (0.9)
IVB	31 (3.3)
Histology (n, %)	
Squamous cell carcinoma	929 (99.2)
Adenocarcinoma	7 (0.8)
Dose of EBRT (n, %)	
45Gy	96 (10.3)s
50 Gy	840 (89.7)
Dose of BT (n, %)	
24 Gy	656 (70.1)
30 Gy	280 (29.9)
Vaginal involvement (n, %)	
Upper third	83 (8.8%)
No involve	853 (91.2%)
chemotherapy	
Yes	936 (100%)
No	0 (0)
EBRT	
3D-CRT	197 (21%)
IMRT/VMAT	739 (79%)
EBRT field	
Pelvis field	924 (98.7%)
Extended field	12 (1.3%)

### Dose evaluated at PIBS

The mean and median summed doses from EBRT and BT at points PIBS, PIBS+ 2 cm, PIBS-2 cm, and A were expressed as EQD2_α/β = 3_ (Table 2). Almost all patients received the complete EBRT dose from the upper border of the vagina to the level of the PIBS. The median doses of EBRT at PIBS+ 2 cm, PIBS and PIBS-2 cm were 50 (45–50) Gy, 50 (45–50) Gy and 0 Gy, respectively. The median total (EBRT+BT) doses in EQD2_α/β = 3_ at PIBS+ 2 cm, PIBS, and PIBS-2 cm were 82.5(52.7–392.1) Gy, 56.2(51.4–82.1) Gy, and 2.6(0.9–7.4) Gy, respectively. The median values of the doses administered at points A1 and A2 were 84.0 (62.3–90.1) Gy and 84.4 (72.7–92.6) Gy, respectively. One center in this study used the dose-volume evaluation system in brachytherapy. However, we used the point A dose-evaluation system to evaluate the BT dose when analyzing the data, so the minimum dose in point A was quite low.

**Table 2:** Summed doses from EBRT and BT in EQD2

	Dose of EBRT Median (min-max)	Total dose (EBRT+BT) Mean ± SD	Total dose (EBRT+BT) Median (min-max)
A1 (Gy,EQD2_α/β = 10_)	50 (45–50)	83.3 ± 2.5	84.0 (62.3–90.1)
A2 (Gy,EQD2_α/β = 10_)	50 (45–50)	83.6 ± 2.5	84.4 (72.7–92.6)
PIBS+ 2 (Gy,EQD2_α/β = 3_)	50 (45–50)	99.4 ± 55.4	82.5 (52.7–392.1)
PIBS (Gy,EQD2_α/β = 3_)	50 (45–50)	57.7 ± 5.2	56.2 (51.4–82.1)
PIBS-2 (Gy,EQD2_α/β = 3_)	0	2.8 ± 1.1	2.6 (0.9–7.4)
ICRU-R (Gy,EQD2_α/β = 3_)	–	79.9 ± 13.6	77.5 (54.8–132.4)
ICRU-B (Gy,EQD2_α/β = 3_)	–	83.6 ± 2.54	84.4 (72.7–92.6)

### Comparison between two groups with different VRL

**Fig. 1:**
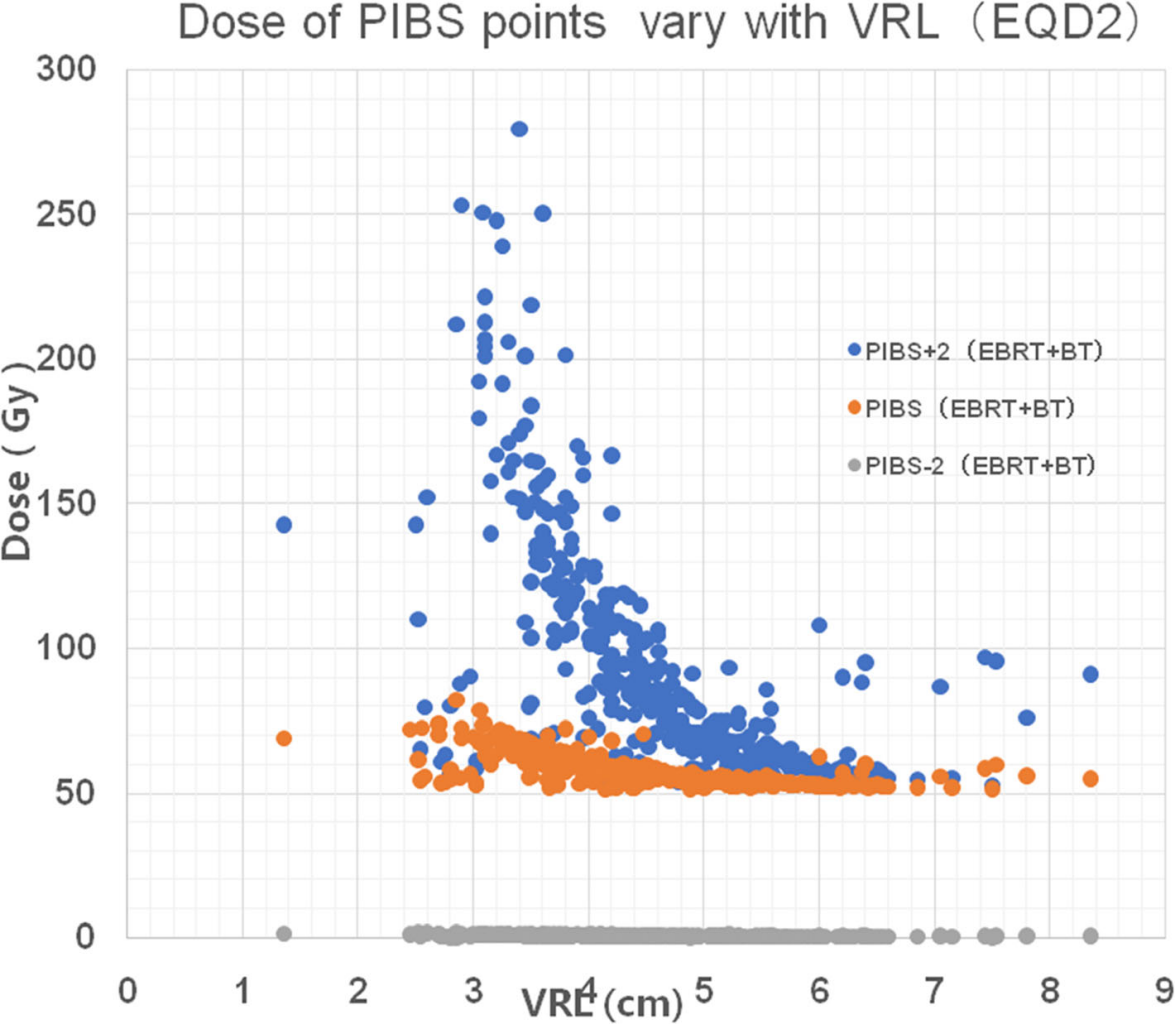
Scatter diagram showing that the doses at PIBS points varied with the VRL. The dose at the PIBS+ 2 cm point changed steeply, while the dose at PIBS-2 cm changed only slightly with increasing VRL. The change in the PIBS dose was moderate

Patients were classified into two groups based on a cut-off VRL of 4.5 cm; one group included patients with a VRL ≤4.5 cm and the other patients with a VRL > 4.5 cm. The mean total dose of EBRT+BT administered at points PIBS+ 2 cm, PIBS, and PIBS-2 cm in the two groups are shown in Table 3. Large variations were noted between the two groups in the total (EBRT+BT) doses in EQD2_α/β = 3_ at these three PIBS levels. The analysis of dose variation at the PIBS points according to the VRL (Fig. 1) showed that a shorter VRL resulted in a higher total dose at point PIBS+ 2 cm and PIBS; in fact, the dose at the PIBS+ 2 cm point showed significant changes with differing VRL, although the dose at the PIBS-2 cm point did not show any marked changes with differing VRL. However, the PIBS point showed only moderate changes with differing VRL. Once the VRL value increased to more than 4.5 cm, the dose value stabilized.

**Table 3:** Total (EBRT+BT) doses at PIBS+ 2 cm, PIBS and PIBS-2 cm according to a VRL cut-off of 4.5 cm

	Total dose (EBRT+BT) Mean ± SD		P
	VRL ≤4.5 cm	VRL > 4.5 cm	
PIBS+ 2 cm (Gy, EQD2_α/β = 3_)	128.5 ± 64.2	68.9 ± 11.9	< 0.01
PIBS (Gy, EQD2_α/β = 3_)	60.7 ± 5.5	54.5 ± 1.8	< 0.01
PIBS-2 cm (Gy, EQD2_α/β = 3_)	0.8 ± 0.3	0.5 ± 0.1	< 0.01
ICRU-R (Gy, EQD2_α/β = 3_)	82.0 ± 14.1	77.6 ± 12.9	< 0.01
ICRU-B (Gy, EQD2_α/β = 3_)	85.0 ± 13.6	79.8 ± 13.2	< 0.01

**Fig. 2:**
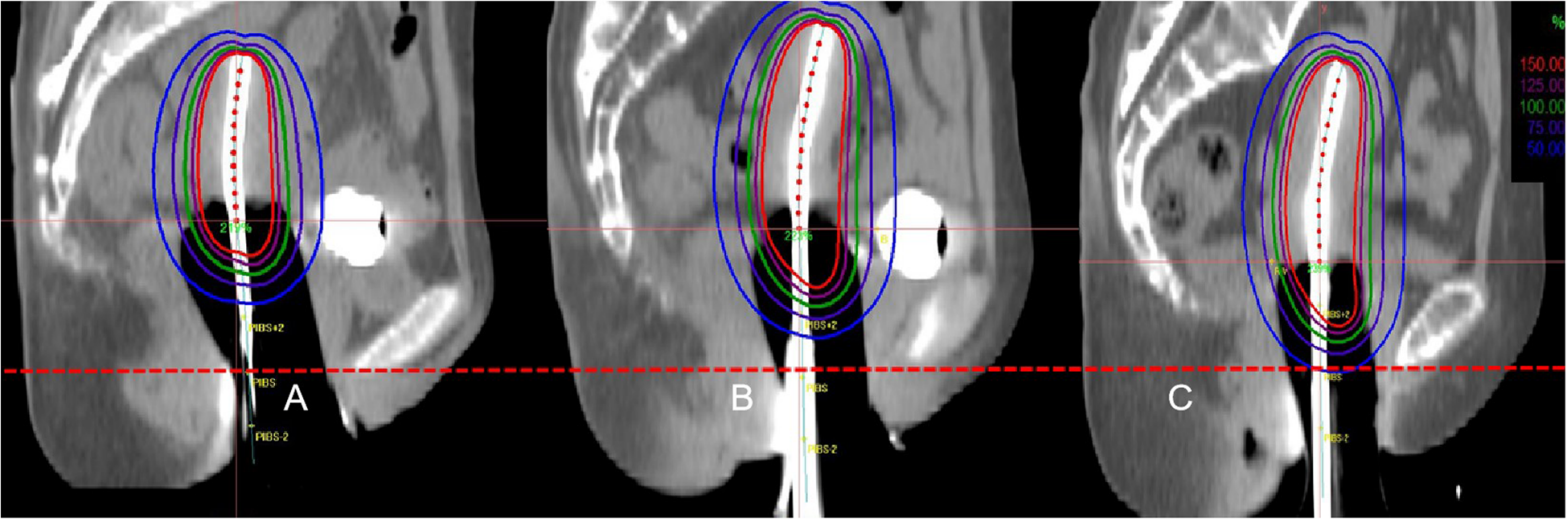
BT CT sagittal view for a patient with tandem and ovoid applicator. The picture shows three cases of BT planning for three patients with different VRLs. **a**: Patient with a VRL of 6.8 cm. **b**: Patient with a median VRL of 4.5 cm. **c**: Patient with a VRL of 3.5 cm, in whom the PIBS+ 2 cm point was located in the high dose area. Red contour: the 150% isodose line. Green contour: the 100% isodose line. Red dashes: level of PIBS points. Yellow dot: PIBS+ 2 cm point. With a shorter VRL, the PIBS+ 2 cm point becomes closer to the vaginal applicator. Definition of the PIBS+ 2 cm is not applicable anymore

Considerable dose variation was seen at both levels. The mean total doses in the VRL ≤4.5 cm and VRL > 4.5 cm groups were 128.5 Gy and 68.9 Gy at point PIBS+ 2 cm, 60.7Gy and 54.5 Gy at point PIBS, and 0.8 Gy and 0.5 Gy at point PIBS-2 cm, respectively. Figure 2 clearly shows that when the VRL = 4.5 cm, the PIBS+ 2 cm point was located between the 75% isodose line and 50% isodose line; when the VRL > 4.5 cm, the PIBS+ 2 cm point was located out of the 50% isodose line; and when the VRL < 4.5 cm, the PIBS+ 2 cm point was located within the 100% isodose line.

### Comparison between two fractions of BT

Patients underwent CT localization at the time of BT fractions 1 and 3, and the dosage values of PIBS, PIBS±2 cm, ICRU-R, and ICRU-B points obtained at BT1 and BT3 were recorded, as shown in Table 4. All the parameters in the two fractions were similar and showed no difference between BT1 and BT3 (*P* > 0.05).

**Table 4:** Comparison of parameters between BT1 and BT3

	BT1	BT3	*P*-value
Oviods distance (cm)	2.8	2.8	0.614
Tandem length (cm)	6.0	6.1	0.105
VRL (cm)	4.5	4.5	0.947
A1 (Gy, EQD2_α/β = 10_)	7.9	7.9	0.637
A2 (Gy, EQD2_α/β = 10_)	8.0	8.0	0.242
PIBS+ 2 cm (Gy, EQD2_α/β = 3_)	12.6	18.3	0.124
PIBS (Gy, EQD2_α/β = 3_)	1.9	2.0	0.478
PIBS-2 cm (Gy, EQD2_α/β = 3_)	0.6	0.7	0.128
ICRU-R (Gy, EQD2_α/β = 3_)	7.4	7.3	0.743
ICRU-B (Gy, EQD2_α/β = 3_)	7.1	6.5	0.064

## Discussion

The dose administered at the target area and the irradiation volume are closely related to the occurrence of radiotherapy-related injury of the rectum and bladder; accordingly, some restrictions have been established for safe dosage amounts of radiation for the rectum and bladder [10, 11]. Similar to the case in bladder and rectal, radiotherapy-induced injury in the vagina may occur at any time within the first 2 years of radiation treatment. A new method for dose evaluation in the vagina using the PIBS points was recently proposed by the EMBRACE study [12]. In the PIBS system, the dose variations at PIBS points are determined by the demarcation of the EBRT border, the extent of vaginal involvement at the time of BT and the VRL [4]. Our findings suggest that this method of assessing the total EBRT and BT dose administered in the upper, middle (PIBS+ 2 cm), and lower (PIBS and PIBS-2 cm) parts of the vagina is applicable even in a multicenter setting. In our cohort, the lower border for the EBRT field was located approximately at the level of the PIBS or below; thus, the vagina above the PIBS level received a radiation dose of 50 Gy from EBRT. Variations in the dose administered to the upper segment of the vagina can be mainly attributed to variations in the BT dose. And at the time of BT, the residual lesions were mainly localized at the level of the vaginal fornix. This implies that the VRL plays an important role in the dose variation at the PIBS.

It is obvious that the VRL value of Chinese patients is much shorter than that reported by studies conducted in other regions. Accordingly, the median VRL among our patients was 4.5 (1.3–8.4) cm, which is considerably different from the VRL 6.1 (1.6–11.0) cm reported in 2013 and 5.4 (1.0–8.3) cm in 2016 on conventional radiographsin by Westerveld et al. [4, 13]. The value of VRL depends on many factors, such as genetic, ethnic, geographical [14, 15], the residual lesions and the position of the vaginal applicator at time of BT. For instance, a large remnant tumor mass after EBRT may decrease the VRL. Radiotherapy (EBRT) may lead to vascular toxicity and fibrosis, rendering the vagina short and narrow [16].. Berger et al. concluded that geometrical shifts of even 1 mm lead to a 25% change in the estimated dose to the vagina [17]. Therefore, BT was performed after EBRT. The vaginal applicator was placed and fixed firmly, as better repeatability among every fraction of BT can reduce dose variations at the PIBS. In our study, statistical analysis showed no significant difference between the doses of the two fractions of BT. In the present study, we selected patients of age ≤ 60 years because age is also a factor that can affect the VRL, as the VRL decreases with age. .

Interestingly, the median doses reported in our series differed from those reported in the series by Westerveld et al. [4, 16] (Table 5). At point PIBS+ 2 cm, the median total (EBRT + BT) doses reported by Westerveld et al. in their 2013 and 2016 studies were 49.6 (32.1–89.6) Gy and 54 (32–109) Gy, respectively, whereas that in our study was 82.5 (52.7–392.1) Gy. Thus, compared to their results, ours showed that the dose at PIBS+ 2 cm was 30 Gy higher. At the PIBS point, the values in Westerveld et al. studies were 36.7 (3.1–68.2) Gy and 41 (3–81) Gy, respectively, whereas that in our study was 56.2 (51.4–82.1) Gy. Thus, the dose at point PIBS in our study was equivalent to the dose reported for the PIBS+ 2 cm point by Westerveld et al. Because the VRL in the Chinese women included in our study was 1.5–2 cm less than that reported for European patients, more volume of the vagina received high-dose radiation. Further, the PIBS-2 cm point represents the level of the vaginal introitus, and the dose values observed here indicate that patients received extremely low doses at this level that did not cause serious radiation injury in most cases. Figure 1 shows that the dose at the PIBS+ 2 cm point varied drastically with differing VRL on account of near of the source. However, dose changes at the PIBS point with variation in the VRL were moderate. So the PIBS point could be considered as a good indicator of the radiation dose applied to the vagina and predictor of late morbidity caused by high-dose exposure of the vagina, especially in patients with a VRL shorter than 4.5 cm.

**Table 5:** Comparison in PIBS points

	Total dose (EBRT+BT) Median (min-max)	
	Westerveld.H	Our Study
PIBS+ 2 (Gy,EQD2_α/β = 3_)	50 (32–90)	82.5 (52.7–392.1)
PIBS (Gy,EQD2_α/β = 3_)	37 (3–68)	56.2 (51.4–82.1)
PIBS-2 (Gy,EQD2_α/β = 3_)	4 (1–46)	2.6 (0.9–7.4)

The above-mentioned findings can be explained by a few points. First, the VRL is the basis of the dose determination using the PIBS system. Second, the type of applicator used may influence the dose received by the vagina. In the multicenter study by Westerveld et al., it was evident that different applicators led to variation in the radiation dose applied at the vaginal wall. For example, the tandem-ovoid applicator normally administers higher doses at the anterior and posterior vaginal walls as compared to the tandem-ring applicators, whereas the latter administers higher doses at the lateral wall of the upper part of the vagina as compared to the former [9]. Third, the application of the point A dose evaluation system, which is different from the dose–volume evaluation system used in studies from other countries, may also influence the vaginal doses received. This is evident from the fact that our patients received a higher vaginal dose as compared with those reported in other studies.

To better understand the correlation of the VRL and dose variation at the PIBS system points, we classified our patients into two groups according to the median value of VRL, which was 4.5 cm. The results indicated significant differences in the dose values for patients with a VRL ≤4.5 cm versus a VRL > 4.5 cm. Thus, for patients with a short (< 4.5 cm) VRL, a greater volume of the vagina was exposed to high doses. This would mean an increased risk of vaginal morbidity in the future.

Our study has a few limitations. First, we used the point A dose-evaluation system to evaluate the BT dose. The dose for the vagina and surrounding OARs may have been lower if we had used the dose–volume evaluation system, especially in cases of large tumors. Second, patients underwent a CT scan only at the time of the first (BT1) and third (BT3) fractions of BT, and using the average values of the BT1 and BT3 for evaluating the other parameters. Therefore, some deviation is possible. Lastly, we only assessed the vaginal dose in cervical cancer using the new evaluation system and did not evaluate the data of incidence of vaginal toxicity. Further studies are required to clarify these issues.

## Conclusions

This observational study indicated that the radiation dose administered at the PIBS points differs significantly with variation in the VRL and that the VRL values for Chinese women were much less than those for European and American women. Therefore, Chinese patients with cervical cancer received higher radiation doses for total EBRT and BT in the upper 2/3 of the vagina when using the PIBS evaluation system. Therefore, it is important to pay attention to the radiation dose received by the vaginal region in order to reduce vaginal radiation injury. The findings of this study provide the basis for the efficient use of PIBS in the evaluation of the vaginal radiation dose in Chinese patients. Further studies are necessary to correlate these parameters with vaginal morbidity.

## Data Availability

The datasets used and/or analyzed during the current study are available from the corresponding author on reasonable request.

## Abbreviations

PIBS: Posterior-inferior border of symphysis
FIGO: International Federation of Gynecology and Obstetrics
EBRT: External-beam radiotherapy
BT: Brachytherapy
3DCRT: 3D-conformal radiotherapy
IMRT: Intensity-modulated radiotherapy
RTOG: Radiation Therapy Oncology Group
3D: Three-dimensional
CT: Computed tomography
HDR: High-dose rate
SIM: Simulation
VRL: Vaginal reference length
ICRU: International Commission on Radiation Units
